# Modulation of salt-induced stress impact in *Gladiolus grandiflorus* L. by exogenous application of salicylic acid

**DOI:** 10.1038/s41598-021-95243-9

**Published:** 2021-08-02

**Authors:** Malik Fiaz Hussain Ferdosi, Amna Shoaib, Salma Habib, Kashif Ali Khan

**Affiliations:** 1 Department of Horticulture, Faculty of Agricultural Sciences, University of the Punjab, Quaid-e-Azam Campus, Lahore, 54590 Pakistan; 2 Department of Plant Pathology, Faculty of Agricultural Sciences, University of the Punjab, Quaid-e-Azam Campus, Lahore, 54590 Pakistan

**Keywords:** Plant physiology, Plant stress responses

## Abstract

Salinity is challenging threats to the agricultural system and leading cause of crop loss. Salicylic acid (SA) is an important endogenous signal molecule, which by regulating growth and physiological processes improves the plant ability to tolerate salt stress. Considering the prime importance of *Gladiolus grandiflorus* (L.) in the world’s cut-flower market, the research work was undertaken to elucidate salinity tolerance in *G. grandiflorus* by exogenous application of SA irrigated with saline water. Results revealed that increasing salinity (EC: 2, 4 and 6 dS m^–1^) considerably altered morpho-growth indices (corm morphology and plant biomass) in plants through increasing key antioxidants including proline content and enzymes activity (superoxide dismutase, catalase and peroxidase), while negatively affected the total phenolic along with activity of defense-related enzymes (phenylalanine ammonia lyase, and polyphenol oxidase activity). SA application (50–200 ppm) in non-saline control or saline conditions improved morpho-physiological traits in concentration-dependent manners. In saline conditions, SA minimized salt-stress by enhancing chlorophyll content, accumulating organic osmolytes (glycine betaine and proline content), total phenolic, and boosting activity of antioxidant and defense-related enzymes. Principle component analysis based on all 16 morphological and physiological variables generated useful information regarding the classification of salt tolerant treatment according to their response to SA. These results suggest SA (100 or 150 ppm) could be used as an effective, economic, easily available and safe phenolic agent against salinity stress in *G. grandiflorus*.

## Introduction

Increasing salinization of water resources and agricultural lands has become the global dilemma of the twenty-first century, which endangers the potential use of soils for crop cultivation. It has been reported that about 85% of the world area is slightly to moderately affected by salinity, while remainder 15% is prone to high salt stress making them unfit for crop cultivation^1^. In Pakistan, ten million thousand hectares is affected and about 5–10 hectares per hour is lost to salinity and/or waterlogging in coastal regions and in the irrigated Indus basin. Data of the previous studies further indicated losses of US$ 27.3 billion per year globally in crop production due to salt-induced land degradation in irrigated areas^2^.

Under current water crises, it is now become imperative to improve alternative agriculture strategies. Utilization of salinized land or salinized water for crop cultivation may be one step towards the Millennium Development Goals that can lead to sustainable agriculture without destroying lands and natural resources^3^. The global flower industry is currently an important sector in the world's economy and Gladiolus is one of the high valued commercial cut flower crop even for small farmers in Pakistan. *Gladiolus grandiflorus* L., is queen of bulbous flowers and one of the high valued commercial cut flower crops in global flower bulb market^4^. The plant is grown in tropical to temperate regions, and is extensively used in flower arrangement, bouquets, beddings of gardens, pot cultures and rockeries due to its outstanding colors, spike, vigor, appearance and long shelf life. In Pakistan, Gladiolus is the market leader in terms of flowers selling and cultivation after roses and tube roses, while it occupies 2000 hectares. Although its cultivation is getting importance among the farmers, sill, the commercial cultivation of Gladiolus in the country is restricted to limited areas of the province mainly due to domestic market for these flowers^5^. Gladiolus production could be promoted on land and water unsuitable for conventional crops. In addition to their production capabilities, they can be used simultaneously for landscape reintegration and soil rehabilitation. Thus cultivation of the Gladiolus crops would be an important intervention in wasteland areas of Pakistan, where the farmers can earn much more by exploiting available land more efficiently.

Although salinity (Na^+^/Cl^−^) toxicity induced oxidative stress by over-accumulation of ROS (reactive oxygen species), which damages cell machinery and associated physiological processes in the plants. Some of morphological evidences include reduction in seed germination rate and plant growth, crippled photosynthetic apparatus, homeostatic events including water uptake, transport, transpiration, and nutrient imbalance all are often correlated with progressing senescence processes or with plant death^6^. The scavenging of ROS depends on both enzymatic (superoxide dismutase, SOD; catalase, CAT, peroxidase, POX etc.) and non-enzymatic components. Salicylic acid (SA), a phenolic growth regulator, is a non-enzymatic antioxidant enzyme, which has gained a special importance among all signaling molecules because of its capability to mitigate the effect of biotic or abiotic stresses by extensive signaling cross-talk with other growth substances present in plants^7,8^. Jayakannan et al.^9^ findings revealed that SA application inhibited the hostile influence of salinity in Arabidopsis by dropping the K^+^ leakage and improving the H^+^-ATPase activity in root tissues, which reduces the Na^+^ accumulation in cytosol by strengthening the Na^+^/H^+^ exchanger at the plasma membrane. SA treatment also reduces lipid peroxidation and may interact with other plant hormones to enhance plant resistance and/or tolerance to salt stress^10^. According to Noreen et al.^11^ under saline and non-saline conditions, SA (200 ppm) promoted the stem length and biological yield via enhancing the photosynthetic rate and carbohydrate metabolism and induced antioxidant enzymes. Ahmed et al.^12^ reported that exogenously applied SA (50 and 100 mM) helped *Vicia faba* in regulating the signaling events under NaCl stress through enhancing uptake of nutrients and other physiological characteristics (proline, glycine betaine and activities of antioxidant enzymes), while positively affected growth, biomass yield and pigment system. SA has been suggested as a potential growth regulator to improve tomato plant under salinity stress through regulating osmotic potential^13^. Recently, Naeem et al.^8^ found foliar SA (0.5 mM) as a significant protectant against salinity stress in tomato by retaining growth and quality traits. To the best of our knowledge, there is no information available so far about the effect of SA on morpho-growth and physio-chemical investigation on *G. grandiflorus* under saline conditions. Therefore, cultivation of Gladiolus in a saline condition and application of SA would play an important role in inducing salt tolerance in plants. The objective of the proposed study was to check the growth, morphology along with key physio-chemical parameters of *G. grandiflorus* irrigated with saline water (2, 4 and 6 dS m^−1^) and foliar sprayed by SA (50, 100, 150 and 200 ppm) doses.

## Results

### Growth performance and corm attributes

**Figure 1:**
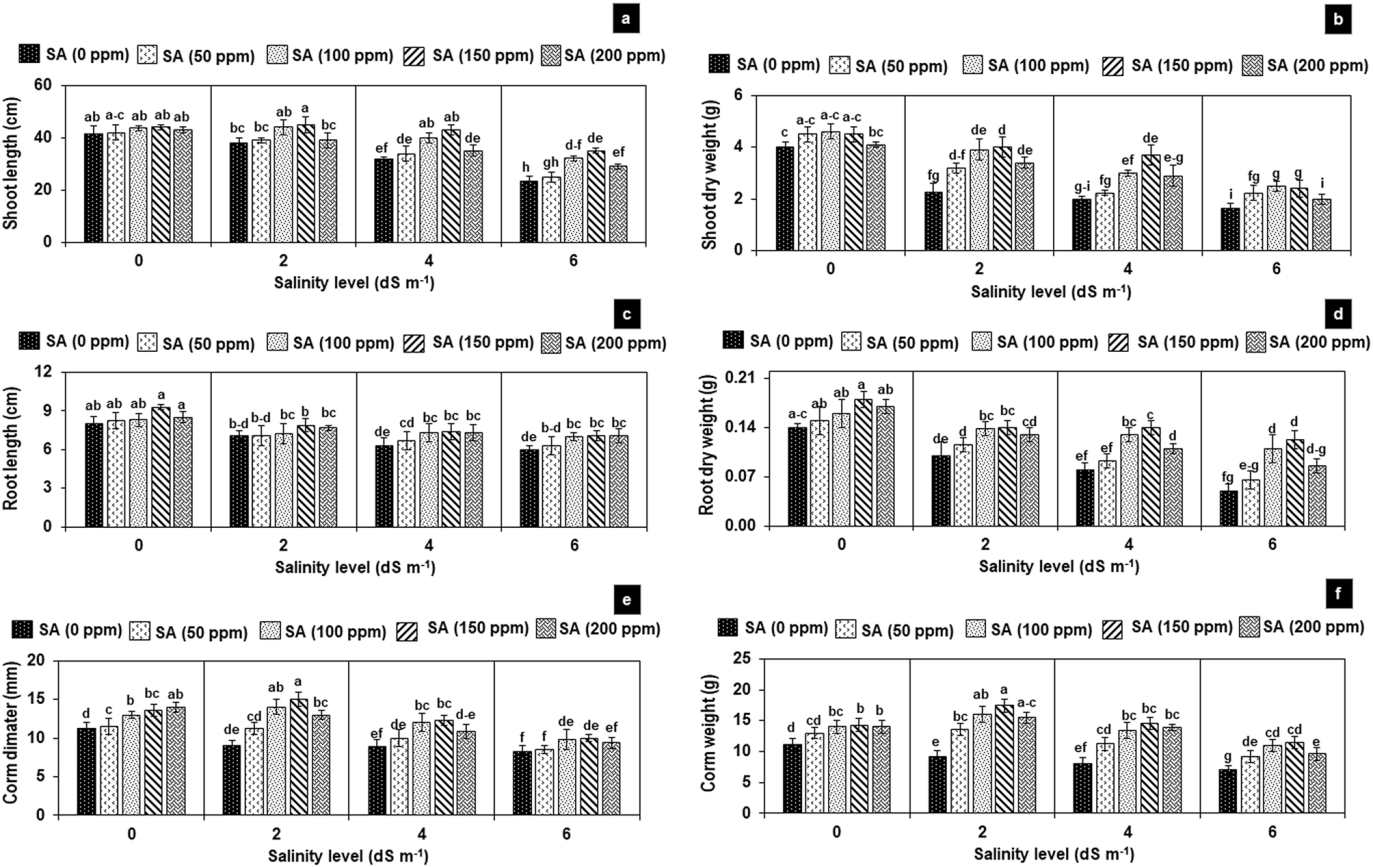
Effect of foliar application of salicylic acid on the growth attributes of *Gladiolus grandiflorus* under salinity stress 90 days after sowing**.** Vertical bars show starred error of mean of replicates. Different letters indicate significant differences (P < 0.05) according to LSD test; the same letter indicates no significant differences between the treatments.

Salinity treatments significantly suppressed the plant growth and development, resulting in a pronounced reduction in the shoot and root growth along with corm indices of 90 days old crop by 20–60% at 2–6 dS m^−1^, while plant length and corm diameter were insignificantly affected at 2 dS m^−1^ as compared to the non-saline control (Fig. 1a–f). Foliar SA displayed insignificant effect on the length and biomass of shoot and root in non-saline treatments (T_2_-T_5_), while significantly improved corm diameter and weight by 20–30% in these treatments as compared to the non-saline control (T_1_) (Fig. 1a–f). However, foliar application of SA significantly improved the biomass and corm attributes, and insignificantly affected the plant’s length in saline-irrigated plants in order of: 100 ≤ 150 ≥ 200 ppm. Thus, the effect of foliar SA improved shoot length by 30–50%, shoot biomass by 50–80%, corm diameter by 40–60% and corm weight by 50–90% in saline-irrigated plants (T_8–10_, T_12–15_ and T_17–20_ ) as compared to their respective non-saline control without SA (T_6_, T_11_ and T_16_) (Fig. 1a–f). The root dry biomass increased more profoundly by 40–150% with application of 100 and 150 ppm of SA at all three levels of salinity as compared to their respective non-SA-treated plants (Fig. 1d).

### Corm morphology

**Figure 2:**
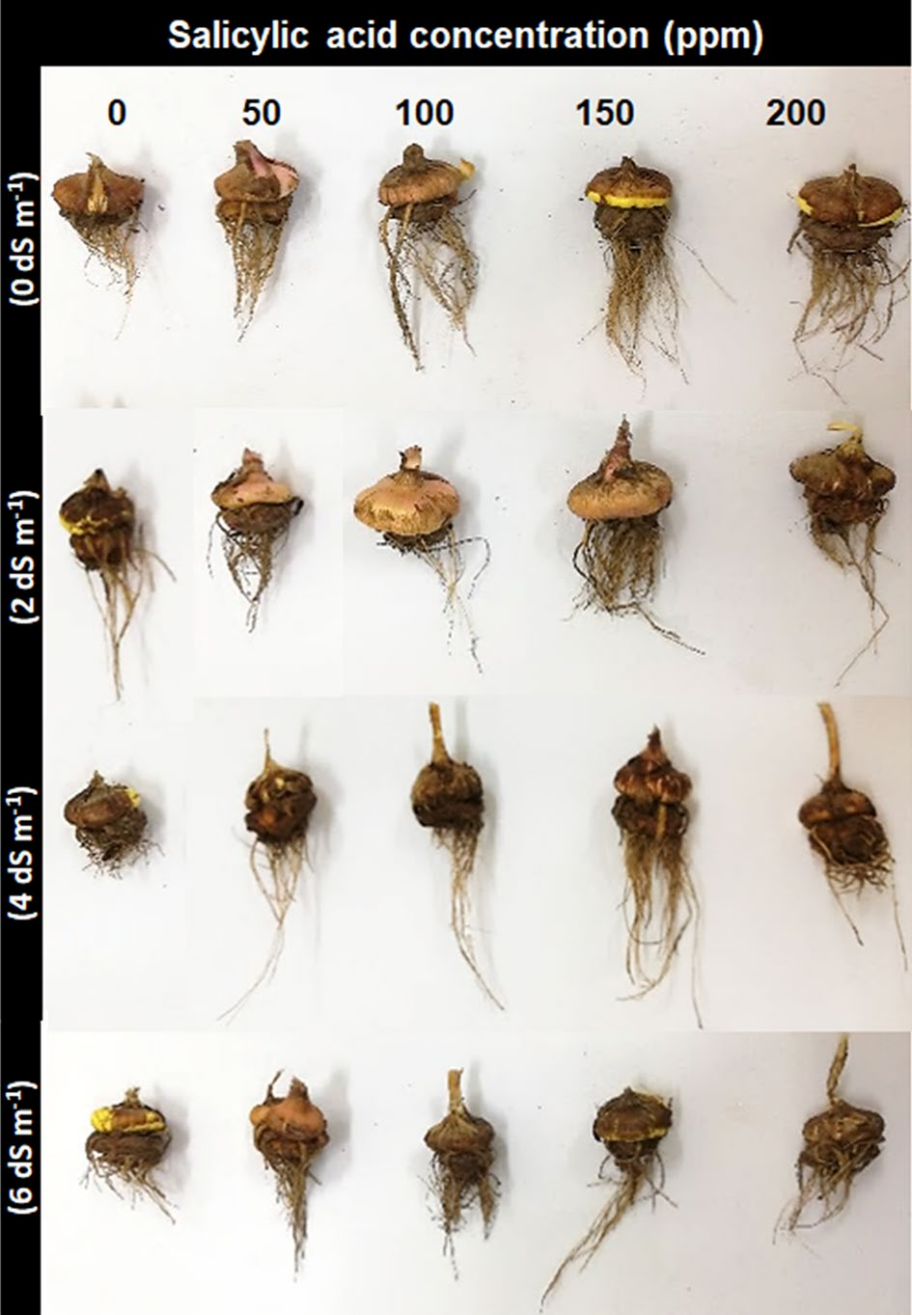
Effect of salicylic acid on corm of *Gladiolus grandiflorus* under salinity stress 90 days after sowing.

In non-saline control treatments, corms were uniform in size, round-shaped, healthy, turgid, and large. They had strong attachment with dense, fibrous and long roots and exhibited cormlets formation. Exogenous application of SA resulted in better root (volume and thickness) and corm morphology (shape and texture) as compared with non-SA-treated plant (Fig. 2). In salt-treated plants (2, 4 and 6 dS m^−1^), the corms were unhealthy, flaccid to turgid, shrunken, smaller in size, irregular in shape, weakly attached to the root. Roots had tiny root hairs and cormlets formation was observed with burning effect on them. However, foliar SA especially concentrations of 100 and 150 ppm exhibited improvement in the attributes of corm, like corms were healthy, roots were longer along with better root hairs, roots was strongly attached to the corm, and there was no burning effect of the corm (Fig. 2).

### Photosynthetic pigments

**Figure 3:**
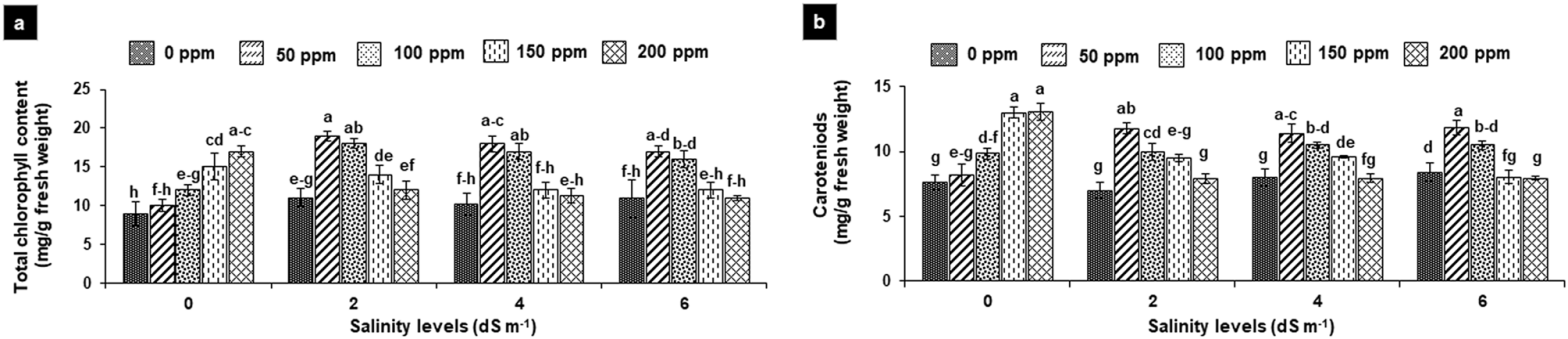
**(a,b)** Effects of foliar application of salicylic acid on the total chlorophyll content (**a**) and carotenoids (**b**) in *Gladiolus grandiflorus* irrigated with saline water. Vertical bars show standard errors of means of three replicates. Different letters indicate significant differences (P < 0.05) according to LSD test; the same letter indicates no significant differences between the treatments.

Total chlorophyll content and carotenoids were insignificantly affected at different levels of salinity (2, 4 and 6 dS m^−1^). Application of different concentrations (50–200 ppm) of SA exhibited inconsistent results in non-salt-treated and salt-treated plants. In non-salt-treated plants, lower concentrations, i.e. 50 and 100 ppm SA did not show any effect on the photosynthetic pigments, but higher concentrations (150 and 200 ppm) significantly increased the said attribute by 70–90%. However, in the salt-treated plant, lower concentrations (50 and 100 ppm) proved more effective in improving the photosynthetic pigments by 30–70%, while higher concentrations exhibited insignificant effect as compared to the non-SA-treated plants (Fig. 3a,b).

### Osmolytes

**Figure 4:**
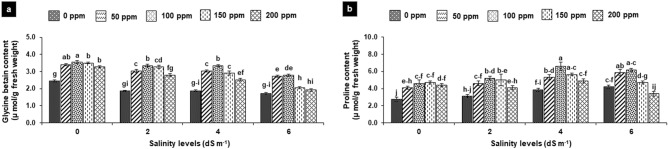
**(a,b)** Effects of foliar application of salicylic acid on the glycine betaine (b) and proline content (b) in *Gladiolus grandiflorus* irrigated with saline water. Vertical bars show standard errors of means of replicates. Different letters indicate significant differences (P < 0.05) according to LSD test; the same letter indicates no significant differences between the treatments.

Salt stress insignificantly decreased glycine betaine content by 22–30% as compared to non-saline control (2.40 µ mol g^−1^ fresh weight). Application of different concentrations of SA effectively improved the glycine betaine in non-saline control by 30–40% and in salt-treated plants by 40–80% as compared to their respective non-SA-treated plants. Moreover, in all treatments, the SA application exhibited that highest increase in the glycine betaine content at 100 ppm followed by at 50 ppm, 150 ppm and 200 ppm of SA (Fig. 4a).

Proline content was insignificantly affected at 2 dS m^−1^, however, increased significantly by 41 and 54% at 4 and 6 dS m^−1^, respectively when compared with non-saline control (2.73 µ mol g^−1^ fresh weight). SA application also showed improvement in proline content of salt-treated and non-salt treated plants. However, application of SA exhibited more pronounced improvement in proline content of the plants at 50–100 ppm concentrations. In non-salt treated plant, foliar SA at 50, 100, 150 till 200 ppm, significantly improved the proline content by 50, 68, 73 and 61%, respectively. The trend was same in salt-treated plant at 2 dS m^−1^, there was a significant increase of 47, 70, 60 and 31% due to SA concentration of 50, 100, 150 and 200 ppm, respectively. At 4 dS m^−1^, SA at 100 proved highly significant as it increased proline content by 71%, followed by improvement of 40% at 50 or 150 ppm. However, at 6 dS m^−1^, only 50 and 100 ppm SA improved the proline content significantly by 40–50% with respect to their respective non-SA-treated treatments (Fig. 4b).

### Antioxidant enzymes

**Figure 5:**
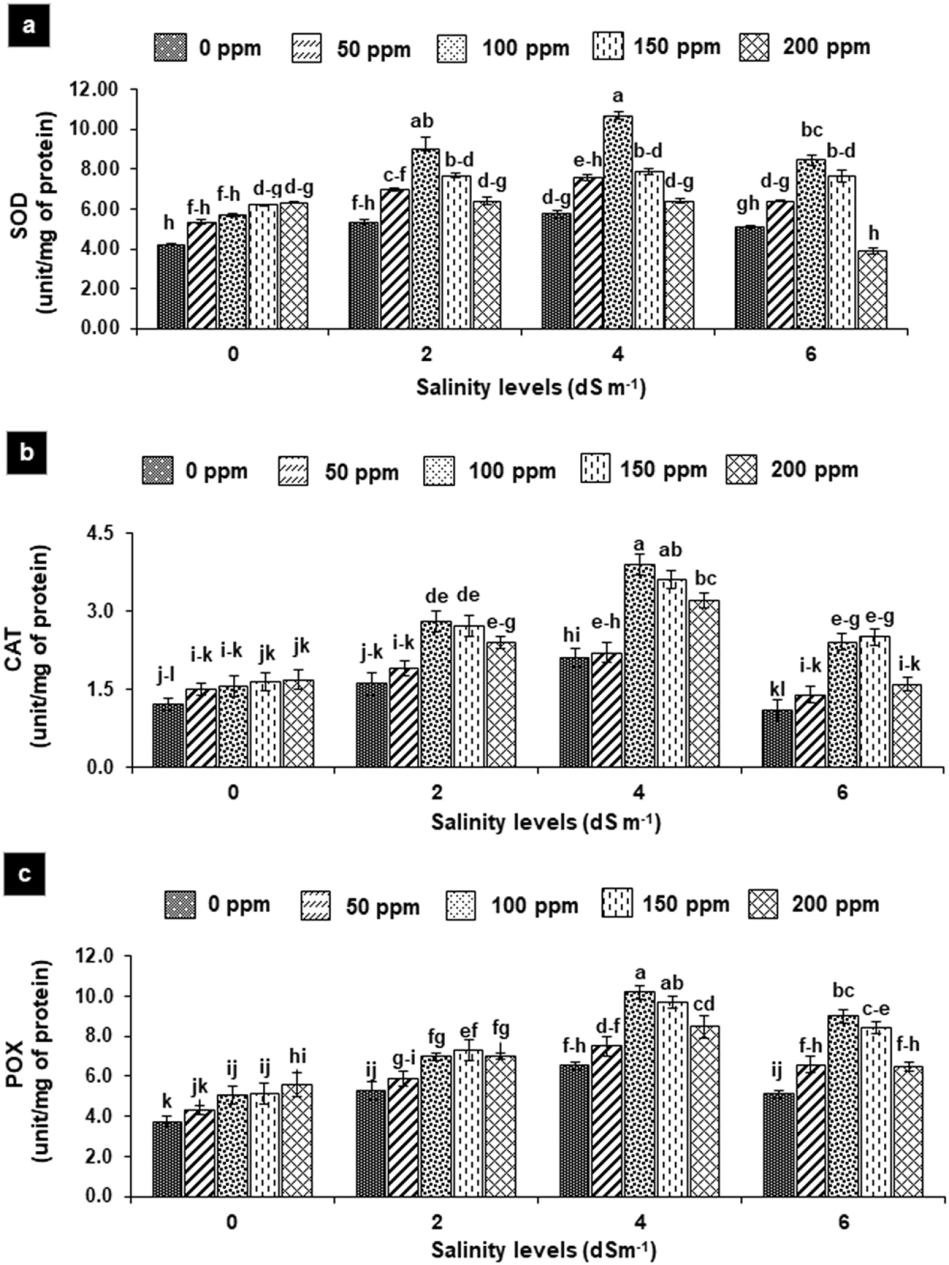
**(a**–**c)** Effects of foliar application of salicylic acid on activity SOD (**a**), CAT (**b**) and POX (**c**) in *Gladiolus grandiflorus* irrigated with saline water. Vertical bars show standard errors of means of three replicates. Different letters indicate significant differences (P < 0.05) according to LSD test; the same letter indicates no significant differences between the treatments.

In salt-irrigated plants, the activity of SOD, CAT and POX elevated significantly, while the highest increased was observed at 4 dS m^−1^ (70–80%) followed by at 2 dS m^−1^ (30–50%) and 6 dS m^−1^ (30–40%) in comparison to non-saline control. Foliar application of SA enhanced enzyme activity followed the similar trend at different concentrations as 100 ≥ 150 > 200 ppm was observed for other physiological traits, while 50 ppm insignificantly affected the said traits in all treatments. Therefore, the activity of enzymes (SOD, CAT and POX) amplified considerably after foliar SA application by 30–50% in water-irrigated plants (non-saline control) as compared to treatments without SA (Fig. 5a–c). Foliar SA in salt-treated plants c5aused the highest increase in the activity of CAT followed by SOD and POX. Therefore, SOD enhanced significantly by 40–70%, 50–80% and 50–60% at 2, 4 and 6 dS m^−1^, respectively compared with non-SA-treated plants. Foliar SA (100–200 ppm) improved the CAT activity by 50–80% 50–90% and 50–130% at 2, 4 and 6 dS m^−1^, respectively. Likewise, SA displayed (100–200 ppm) improvement of 30–40%, 30–50%, and 30–80% in POX activity under saline condition (Fig. 5a–c).

### Effect on total phenolic and defense enzymes

**Figure 6:**
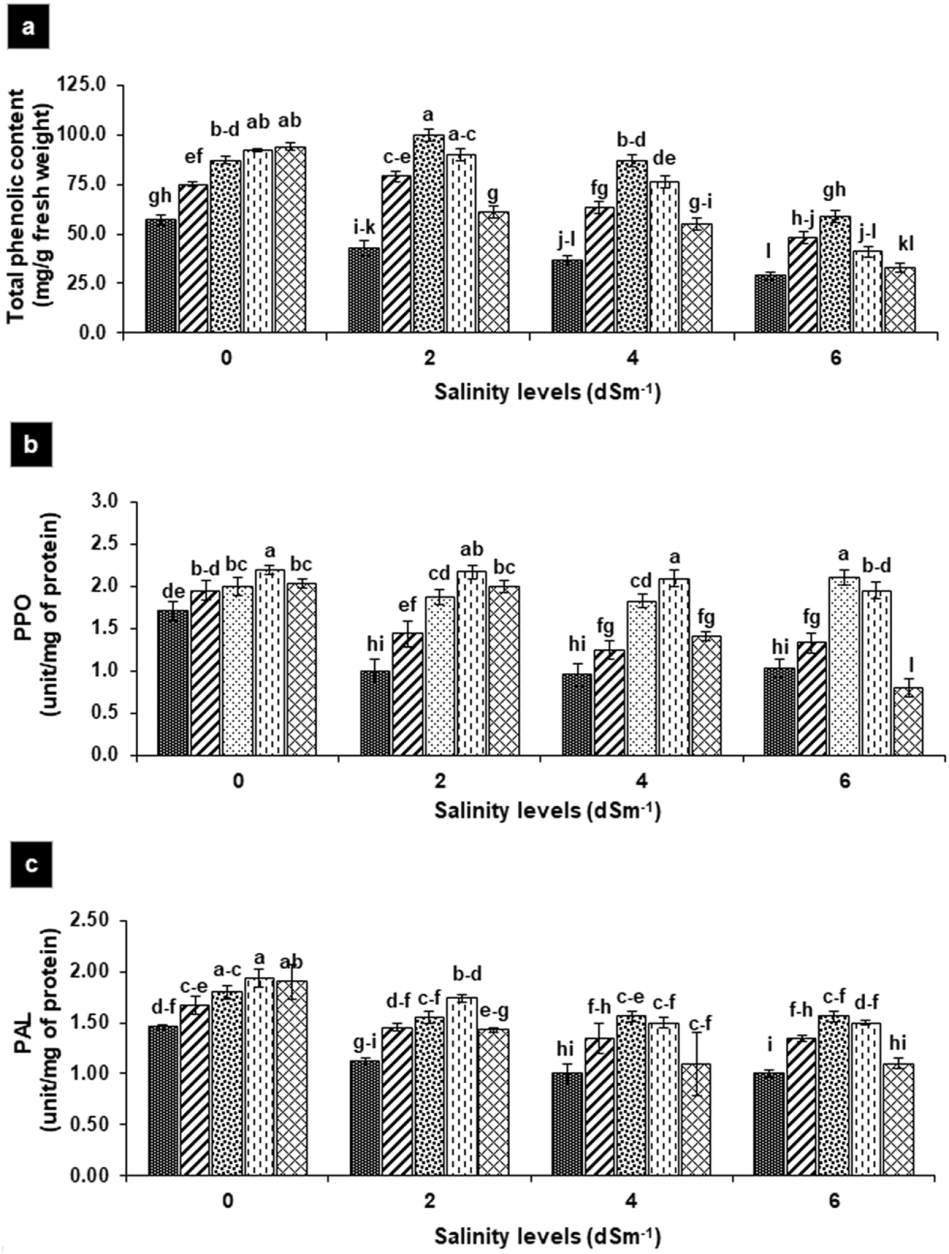
**(a**–**c)** Effects of foliar application of salicylic acid on total phenolics (**a**), PPO activity (**b**) and PAL activity (**c**) in *Gladiolus grandiflorus* irrigated with saline water. Vertical bars show standard errors of means of three replicates. Different letters indicate significant differences (P < 0.05) according to LSD test; the same letter indicates no significant differences between the treatments.

Total phenolic were decreased significantly by 25, 35 and 47% with rising salinity level 2, 4 and 6 dS m^−1^, respectively as compared to non-saline control (57 mg g^−1^ fresh weight). SA accelerated the total phenolic by 30–70% in non-salt treated plants in concentration-dependent manners, the maximum total phenolic were present at 200 ppm SA. However, in salt-treated plants, 100 and 150 ppm of SA revealed the greater improvement of 100–140% at 2 and 4 dS m^−1^, respectively. At higher salinity level (6 dS m^−1^), increasing SA level did not increase the said attributes except at 100 ppm, where improvement was 30% with respect to non-SA-treated plants (Fig. 6a).

Like total phenolic content, the activity of PPO decreased significantly by 40% in salt-treated plants, by contrast, it was raised by 40–117%, 30–120% and 30–100% at 2, 4 and 6 dS m^−1^, respectively after foliar application of SA (50–200 ppm). Likewise, activity of PAL decreased significantly by 30% in salt-treated plants as compared to the non-saline control, while significantly improved by 30–60% after foliar SA (50–150 ppm) with respect to their respective non-saline control. In case of water-irrigated plants, PAL and PPO activity significantly enhanced up to 30% only at higher concentration (150–200 ppm) as compared to non-saline control (Fig. 6b,c).

### Valuation of salt tolerant treatment by PCA

**Figure 7:**
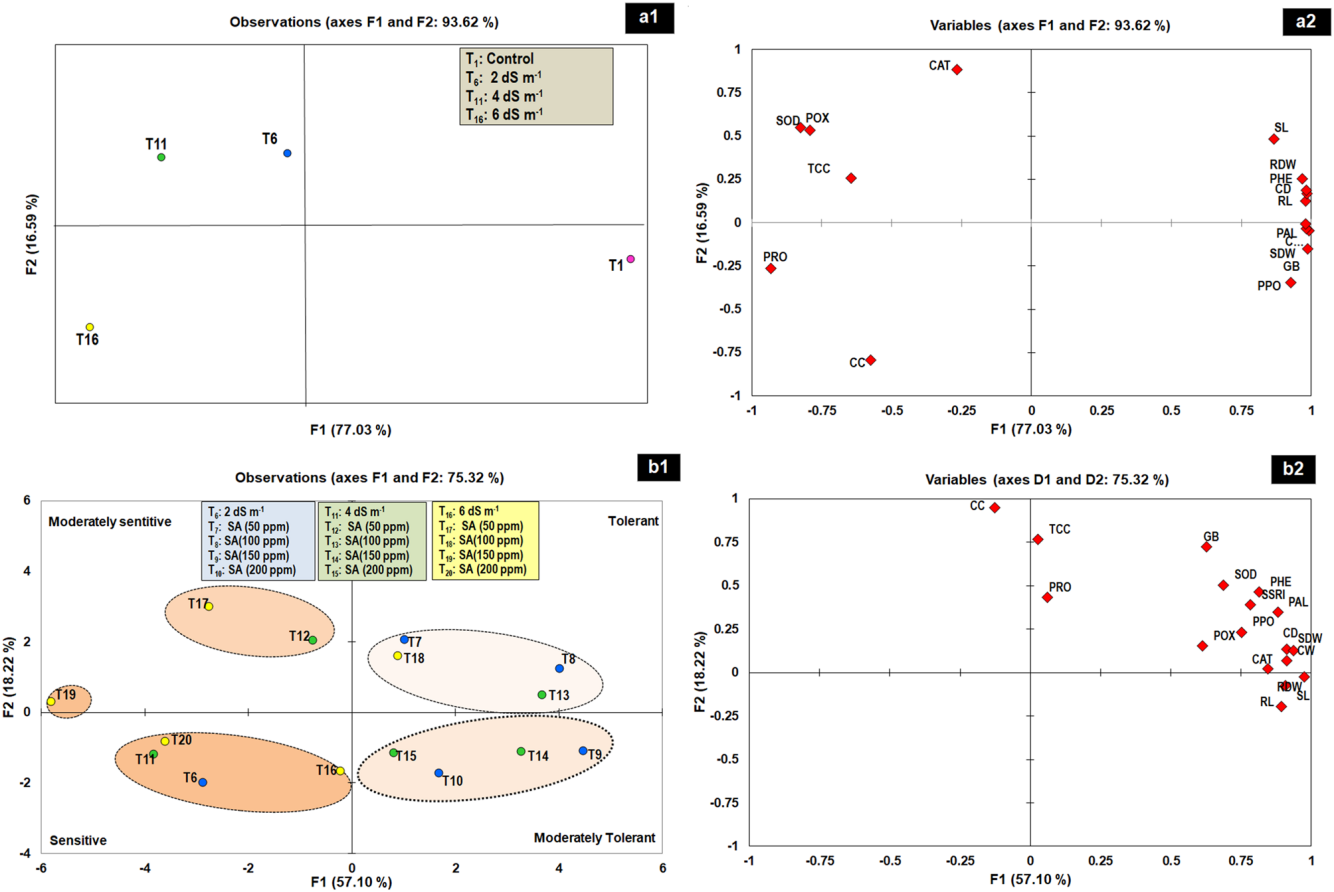
(**a,b**) Biplot representation of the results of principal component analysis (PCA) describing effect of salinity and salicylic acid (SA) (**a**) Treatments response to salinity stress (**a**_**1**_) based on measured properties of the plant (**a**_**2**_); classification of treatments into different salt tolerant groups (salt sensitive, low, moderate, and high salt tolerant) (**b**_**1**_) based on all the morpho-physiological parameters (**b**_**2**_). *SL* shoot length; *SDW* shoot dry weight; *RL* root length; *RDW* root dry weight; *CD* corm diameter; *CW* corm weight; *TCC* total chlorophyll content; *CC* carotenoids; *Pro* proline; *GB* glycine betaine; *PHE* total phenolics; *SOD* superoxide dismutase; *CAT* catalase; *POX* peroxidase; *PPO* polyphenol oxidase; *PAL* phenylalanine ammonia lyase.

**Figure 8:**
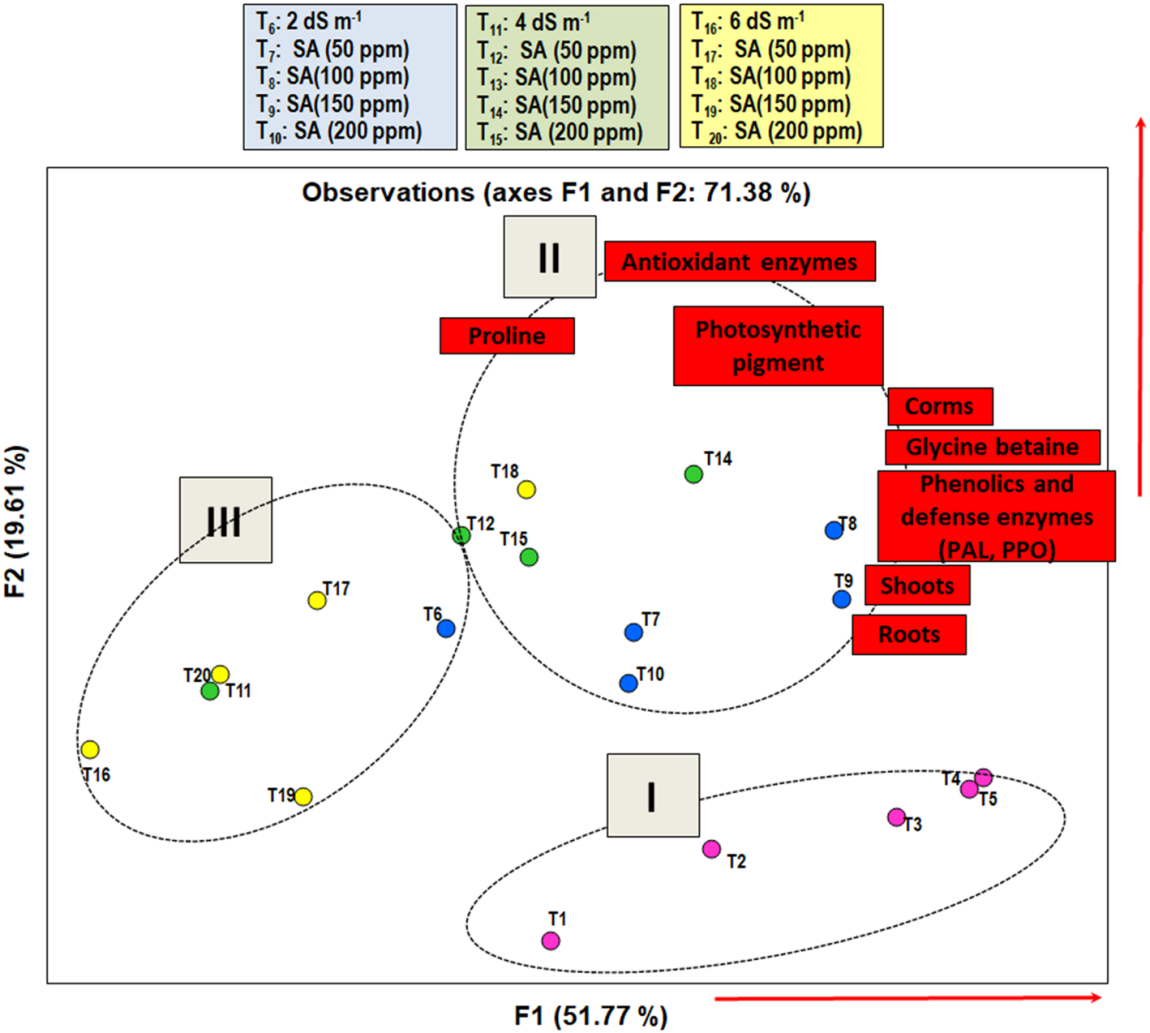
Principal component analysis (PCA) describing effect of salinity and salicylic acid in all 20 treatments in *Gladiolus grandiflorus* and their relationship with the morpho-physiological parameters.

PCA was performed to identify salt tolerant treatment using the principal components of morpho-physiological parameters of Gladiolus in response to salt stress and salicylic acid. PCA analyses with all 16 traits of Gladiolus displayed a good separation between treatments, both in salinity and salicylic acid conditions (Fig. 7a1,a2,b1,b2). The first two principal components (PCs) accounted for 77% and 16% of the total variation among the 4 treatments separated them in three groups (1) treatments in non-saline control conditions positioned right side (2) treatments in low (T_6_: 2 dS m^−1^) to moderate salinity (T_11_: 4 dS m^−1^) at upper left side and (3) treatments in high salinity (T_16_: 6 dS m^−1^) at lower left side of the biplot (Fig. 7a1,a2). After application of salicylic acid in salt-stressed plants, four groups were observed among 15 treatments (Fig. 7b1), where PC1 and PC2 collectively explained more than half of the variation (Fig. 7b2) and contributed greater importance in the separation of treatments into different categories. Treatments showing highest values for the measured morpho-physiological parameters for PC1 and PC2, located in the upper-right corner of the biplot, were considered as highly salt tolerant treatments (T_7_ and T_8_: 2 dS m^−1^ with 100 and 150 ppm SA; T_13_: 4 dS m^−1^ + 100 ppm SA and T_18_: 6 dS m^−1^ + 100 ppm SA). Treatments with moderate values for PC1 and PC2, located in the lower right of the graph, were considered as moderately salt tolerant (T_9_ and T_10_: 4 dS m^−1^ with 150 and 200 ppm SA; T_14_ and T_15_: 6 dS m^−1^ with 150 and 200 ppm SA). In contrast, genotypes showing the low values of the measured morpho-physiological parameters for PC1 and PC2 fall in the upper and lower left portion of the graph and were considered as moderately salt sensitive to sensitive. Moderately sensitive treatments included T_12_: 4 dS m^−1^ + 50 ppm SA; T_17_ and T_19_: 6 dS m^−1^ with 50 and 150 ppm SA. Sensitive treatments (T_6_: 2 dS m^−1^; T_11_: 4 dS m^−1^ and T_16_: 6 dS m^−1^) included all the plants exposed to salt stress at low, medium and high level without SA application. However, T_20_: 6 dS m^−1^ + 200 ppm SA was also placed in sensitive group. Figure 8 depict the analysis of all 20 treatments and all 16 parameters, where again three groups were observed (1) comprised of all treatments well-irrigated with water and provided foliar SA; (2) contained all treatments irrigated with low and medium salinity levels along with application of different concentration of SA and (3) consisted of treatments received salt stress only along with treatment at high level of salinity provided foliar 50, 150 and 200 ppm of SA. These results clearly revealed that application of SA was more effective at low and medium levels of salinity, while at higher level of salinity only 100 ppm of SA showed effectiveness against salt tolerance in Gladiolus plants. Furthermore, Fig. 8 also showed significance of all 16 growth-physiological attributes in a salt tolerance in group 2 treatments.

## Discussion

Considering importance of biosaline agriculture as well as *Gladiolus*, the current study was carried out to assess the morpho-growth and physio-biochemical alterations in *G. grandiflorus* irrigated with saline water (2, 4 and 6 dS m^−1^), provided with foliar application of salicylic acid (SA) (50–200 ppm). Salinity stress led to reduction in the investigated attributes of shoot, root (growth and biomass) and corm (diameter and weight) by 30–60%, while the negative consequences of salinity was more noticeable at 4 and 6 dS m^–1^. Instability in the nutrients uptake generally induces membrane dysfunctioning and metabolic activity attenuation, which accelerate the biomass inhibition in plants as a result of NaCl toxicity^14^. Likewise, weakening, deforming, discoloration in corms could be outcome of osmotic stress caused by the increase in concentration of salts^15^. Foliar application of SA significantly improved plant growth indices in non-salt treated plants by 20–30% at 150 and 200 ppm as compared to plants without SA. Nevertheless, in salt-treated plants, 100 and 150 ppm of SA showed promising improvement of 50–150% in the growth indices of plant and corm compared with corresponding treatments without SA. Previously, exogenous application of SA found effective in increasing plant growth under both non-stressed and stressed conditions^16^. Li et al.^17^ and Ma et al.^18^ also reported a neutralizing effect of foliar application of SA under salinity, whereas high biomass production after SA application could be attributed to variation in allocation of biomass to different organs, which may be crucial to the success of a seedling to adapt to a new environment^19^. Li et al.^17^ and Misra et al.^20^ have shown that recovery in plant dry biomass in response to SA treatment has been related to the induction of protective role of membranes. It has also been reported earlier that salinity restricted plant growth primarily through disturbing growth traits of the root, while improvement in root biomass may help to sustain vigorous shoot growth and corm indices conceivably by salt dilution or salt exclusion during uptake, restraining the building up of the harmful concentration of Na^+^ ions in the these plant parts consequently better shoot growth and corm attributes^14^. Hence, higher root biomass after SA application revealed greater significance of root attributes in identifying salt-tolerant treatments. Furthermore, 100 and 150 ppm of SA induced more salinity tolerance in the plants followed by 50 and 200 ppm specifically at low (2 dS m^−1^) and medium (4 dS m^−1^) levels of salinity, while with increase in salinity (6 dS m^−1^), 150 and 200 ppm proved unsuccessful against salt tolerance. Khan et al.^21^ study affirmed dose-dependent response of SA, where low and high concentrations stimulated or hindered the plant function. It might be ascribed to SA induced intensification Cl^−^ ions in plants on account of increase in oxidative damage during high salt concentration. Accumulation of toxic anion (Cl −) in the leaves may possibly have compromised the photosynthetic machinery, which negatively alters growth^22^.

Total chlorophyll content and carotenoids were insignificantly affected at different levels of salinity (2, 4 and 6 dS m^−1^). It has been documented that chlorophyll level either increased or unchanged in salt-tolerant plants, whereas it decreased in salt-sensitive plants^23^. Foliar application of SA at higher concentrations (150 and 200 ppm) increased photosynthetic pigments by 70–80% in non-salt-treated plants, whereas, SA at low concentrations (50 and 100 ppm) improved photosynthetic pigments 30–80% in salt-treated plants. Maintenance of the stomatal conductance by the application of SA plays an important role in maintaining photosynthetic activity^24^. Several investigations indicated that SA is a strong regulator of photosynthesis and chlorophyll composition in leaves by influencing chlorophyll content, carotenoid composition, and stomatal closure^25^.

When the plant is under salinity stress, osmotic tolerance occurs by osmotic regulation process through accumulation and then movement of osmolytes via stomatal opening which influences water movement between the cells^26^. Glycine betaine (GB) and proline (Pro) are the most important organic osmolytes and efficient compatible solutes^19^ and their accumulation may specify adaptation of plants to changes in the external osmotic potential under salt stress^27^. In the present study, salt stress (2–6 dS m^−1^) insignificantly decreased GB content and significantly increased Pro content as compared to non-saline control. However, SA at 50–150 ppm significantly increased GB and Pro levels under saline as well as non-saline control, while 200 ppm insignificantly affected the attributes under saline condition. The result suggested a positive role of GB and Pro against salinity. Khan et al.^28^ found involvement of GB in mitigation of salinity-inhibited photosynthesis and plant growth in *Vigna radiata* by SA (at 0.5 mM). Likewise, induction in the activity of proline biosynthesis enzymes has been attributed to occur through NDR1-dependent signaling pathway and was shown to modulate calcium (Ca)-mediated oxidative burst defense response in plants after SA application^29^. The net positive affect of GB and Pro accumulation under high salinity may be correlated with activation of host defense machinery against salt-induced stress in the plants.

ROS hijacked host defense machinery within plant cells due to irregularities in the electron transport chain and accumulation of photoreducing power, and enzymatic antioxidants like SOD, POX and CAT under salt-induced stress. These enzymes have been well-known antioxidants that alleviating salt stress-induced oxidative damage by up-regulating their activity^30^. In current work, generally activities of enzymes increased at low (2 dS m^−1^) reached maximum at medium (4 dS m^−1^) levels of salinity then increased was less pronounced at higher (6 dS m^−1^) level. Enzyme activity enhanced at higher concentration (150 and 200 ppm) of SA in non-saline control, and at low to medium concentrations (50 to150 ppm) of SA in saline treatments, displayed a crucial role of SA in modulating the cell redox balance and protecting the given plants from oxidative damage^7^. Several studies have demonstrated that salt-tolerant species show increased antioxidant enzyme activities and antioxidant contents in response to salt stress, whereas salt-sensitive species fail to do so^31^. Increase in SOD activity may explore the efficient role of SOD in ROS scavenging process in Gladiolus especially at low and medium levels of salinity. The stimulation of POX and CAT suggested that these enzymes are important in the detoxification of H_2_O_2_ in plant seedlings under salinity stress^32^. These results are in agreement with that reported by El-Esawi et al.^25^, accordingly SA decreased NaCl toxicity and enhanced antioxidant enzymes activities (SOD, APX, and CAT) in *Rosmarinus officinallis*. Moreover, exogenous SA application at low concentration (0.1–0.5 mM) proved effective in increasing photosynthesis, growth and various physiological and biochemical processes, whereas higher concentrations (> 1 mM) caused stress under saline condition^33^.

The total phenolic decreased with increased in salinity (2–6 dS m^−1^) as compared to non-saline control probably be due to their sensitivity to saline^34^. Foliar SA enhanced the total phenolic in non-saline control and in salt-affected plants by 100–140%, which may assist the plant to lighten the salinity-induced oxidative stress as phenolic content contributes in absorbing and neutralizing free radicals, quenching singlet oxygen, and decomposing peroxides^35^. These compounds also act as the intermediates in the phenylpropanoids pathway and play important roles in flavonoid production and lignin biosynthesis. It has been documented that elevation of phenolic in rosemary subjected to salinity (2–4 dS m^−1^ NaCl) and SA sprays (50–100 ppm) may provide the survival strategies of plants under salt stress conditions. Like total phenolic content, activity of PPO and PAL decreased at different levels of salinity. These results propose the existence of a synchronized response between PAL, PPO, and the concentration of total phenolic under salinity stress. Previously, reduction in PPO activity under water stress condition was correlated with better stress tolerance^36^, while PAL activity decreased in onion under salt stress was correlated with the limited ability of the plants to produce stress-related secondary metabolites^32^. Application of SA either in non-saline control or salt-treated plant increased the PPO and PAL activity may indicate enhanced tolerance in Gladiolus by increasing in phenolic content and other defense-related secondary compounds associated with tolerance^37^.

PCA is the most frequently utilized multidimensional method to classify salt-tolerant treatments, and to identify the key variables and their pattern of correlations in salinity tolerance^14^. PCA has been formerly utilized to classify salinity tolerance in rice^14^, almond^38^; and corn^39^. In the present study, PCA analysis clearly separated salt-tolerant and salt-sensitive treatments based on all 16 physio-growth traits and their SSRI. PCA plots revealed positive correlation of all traits among themselves located in the same direction of the plot, indicating that impact of salinity on these parameters. Further, it was observed that SA improved salinity tolerance and increased plant biomass particularly at 2 and 4 dS m^−1^ NaCl by enhancing chlorophyll content, accumulating osmolytes and total phenolic, boosting activity of defensive (PAL and PPO) and antioxidant enzymes (SOD, CAT and POX). Higher concentrations of SA did not induce tolerance in the plants at a high level of salinity.

## Methods

### Experimental design

The pot experiment was carried out at the experimental area of Faculty of Agricultural Sciences, University of the Punjab, Quaid-e-Azam Campus Lahore (31°32' N latitude and 74°20' E longitudes), during January-April, 2018. The experiment of 20 treatments with 60 pots was kept in a completely randomized design. Each treatment was applied in three separate repetitions with 4 corms in each replicate. Treatments of the experiments are presented in Table 1.

**Table 1:** Experimental treatments.

Salinity levels (dS m^−1^)	Salicylic acid concentration (ppm)				
	0	50	100	150	200
0	T_1_	T_2_	T_3_	T_4_	T_5_
2	T_6_	T_7_	T_8_	T_9_	T_10_
4	T_11_	T_12_	T_13_	T_14_	T_15_
6	T_16_	T_17_	T_18_	T_19_	T_20_

### Growth conditions and treatments applied

During the growing season, the average minimum and maximum temperatures were 15 °C and 30 °C, and the relative humidity was between 50 and 70%. The loamy soil used for the experiment comprised of 45.50% sand, 23.80% clay and 30.70% silt (saturation: 36%; pH: 7; electrical conductivity: 0.1 dS m^−1^; organic matter: 0.85%; calcium (Ca^++^) + magnesium (Mg^++^): 1.11 meq L^−1^; sodium (Na^+^):5.5 meq L^−1^ and chloride (Cl^­^): 0.4 meq L^−1^).

For the experiment, the plastic pots (30 cm width × 46 cm length) were filled with air-dried soil (13 kg pot^−1^) and healthy corms (4 pot^−1^) of uniform size *G. grandiflorus* were sown. Corms/seeds of *G. grandiflorus* (Polar Bear) were obtained from a commercial source (Greenwork, Lahore, Pakistan). Saline water of each EC level (2, 4 and 6 dSm^−1^) was applied to soil saturation capacity at the time of sowing and later as per the crop requirement. *G. grandiflorus* was cultivated using guidelines provided by Directorate of Floriculture, Govt. of the Punjab, Pakistan. NPK was applied after first irrigation at the time of sowing. For Gladiolus, full basal recommended dose of fertilizer N:P:K (120:120:100 kg hac^−1^) was applied by using source urea, diammonium phosphate (DAP) and solo sulphate of potassium (SOP). The amount of NPK was calculated according to soil in pots (13 kg pot^−1^) for all treatments and mixed in distilled water to make a solution for per pot (1.5 L). Salicylic acid (SA; 2-hydroxybenzoic acid) was initially dissolved in 1000 µL dimethyl sulfoxide and concentrations of 50, 100, 150 and 200 ppm were made up with distilled water. A foliar spray of SA was applied twice, as the first spray was applied at 45 DAS, and the another spray was on 5 days later. At each time, SA was uniformly applied to the plants in the early morning and in afternoon, using an atomizer. Tween-20 (0.05%) was included in the spray application as a surfactant at the time of applications.

### Physio-biochemical assays

To estimate physiological and biochemical variations, leaf samples were collected randomly in triplicate after 10 days after fist spray application of SA. These tests were performed in following ways.

### Chlorophyll and carotenoid content

Total chlorophyll content and carotenoid contents were determined according to the method of Lichtenthaler and Buschmann^40^. Leaves (0.1 g) from each treatment were homogenized with 80% ethanol and centrifuged for 5 min at 10, 000 rpm. Absorbance of supernatant was determined at 470, 645 and 663 nm by UV spectrophotometer (BioTek Epoch microplate spectrophotometer), and calculated using the following expressions^40^.

$$\text{Chlorophyll} a (\text{mg}/\text{g FW})=[0.0127 (\text{OD }663)-0.00269 (\text{OD }645)(\text{V}/\text{W})]$$

$$\text{Chlorophyll }b (\text{mg}/\text{g FW})=[0.0229 (\text{OD }645)-0.00468 (\text{OD }663)(\text{V}/\text{W})]$$

$$\text{Total chlorophyll }(\text{mg}/\text{g FW})=[(20.2\times \text{OD}645)+(8.02\times \text{OD}663)(V/(1000\times W)]$$

$$\text{Carotenoids}=[(1000\text{ A}470-3.27 \left(\text{chlorophyll a}\right)-104 \left(\text{chlorophyll b}\right)]/229$$

### Proline content

The leaf sample (0.2 g) was homogenized in 5 mL of 3% sulfosalicylic acid, and reacted with the 2 mL of acid ninhydrin and 2 mL of glacial acetic acid in a test tube for one hour at 100 °C. Reaction was terminated in an ice bath and then the mixture was extracted with 4 mL of toluene. The chromophore containing toluene was aspirated at room temperature and absorbance was taken at 520 nm using toluene as a blank^41^.

### Glycine betaine content

Grieve and Grattan^42^ method was used to check glycine betaine (GB). The leaf sample was crushed in 5 mL toluene-water mixture (0.05% toluene), centrifuged at 10,000 rpm for 10 min and the supernatant was mixed with 1 mL of 2 N H_2_SO_4._ Then 0.5 mL of this mixture and 0.2 mL of Potassium tri-iodide (KI_3_) solution was mixed. The contents were then cooled and mixed with 6 mL of 1–2 dichloroethane (cooled at − 10 °C) mixture. The two layers formed in the mixture, the upper aqueous layer was discarded and the optical density of the organic layer was measured at 365 nm.

### Total phenolic contents

Sample (0.1 g) of the plant material was homogenized with 10 mL ethanol (80%) and was centrifuged at 10,000 rpm for 10 min. The supernatant was collected and again centrifuged. Alcoholic aliquot (1 mL) was mixed with 1 mL of 20% sodium carbonate and later 0.5 mL of Folin-phenol reagent was added. It was boiled for 10 min at 100 °C in water bath. The final volume was made up to 20 mL with distilled water and absorbance of the sample was noted at 660 nm on UV spectrophotometer^43^.

### Enzyme assays

To assess the activity of antioxidant enzymes, 0.1 g of leaf was ground in 5 mL of 0.1 M phosphate buffer and was centrifuged at 1200 rpm for 10 min and supernatant was used for enzyme assays. Super oxide dismutase (SOD) activity was assayed according to the method of Giannopolitis and Ries^44^ by determining the enzyme ability to inhibit the photochemical reduction of nitroblue tetrazolium (NBT). The reaction mixture (3 mL) contained 130 mM methionine, 750 µmol L^−1^ NBT, 100 µmol L^−1^ EDTA, 20 µmol L^−1^ riboflavin and 50 µL enzyme extract was kept under a light for 15 min, and finally measured spectrophotometrically at 560 nm. For catalase (CAT), a mixture containing 50 mM phosphate buffer (pH 7.0), 0.3% H_2_O_2_ and 100 µL enzyme extract was assessed for absorbance at 240 nm at the intervals of 30 s^45^. Peroxidase (POX) activity was determined through protocol of Kumar and Khan^46^. The reaction mixture of POX contained 2 mL of 0.1 M phosphate buffer (pH 6.8), 1 mL of 0.01 M pyrogallol, 1 mL of 0.05 M H_2_O_2_ and 0.5 mL of enzyme extract. The solution was incubated for 5 min at 25 °C after which the reaction was terminated with addition of 1 mL of 2.5 N H_2_SO_4_. The amount of purpurogallin formed was determined by measuring the absorbance at 420 nm. For polyphenol oxidase (PPO)^47^, the reaction mixture consisted 100 µL enzyme extract and 1.5 mL of 0.1 M sodium phosphate buffer (pH 7.0). The reaction started when 200 µL of 0.01 M catechol was added. The changes in the absorbance were recorded at 30 s intervals for 3 min at 495 nm. Phenyl ammonia lyase (PAL) assessment was carried out in a reaction mixture (0.4 mL of enzyme extract, 0.1 M sodium borate buffer of pH 8.8 and 0.5 mL of 12 mM l-phenylalanine) incubated for 1 h in light at 25 °C followed by termination of reaction by incubating mixture at 47 °C for 10 min. The absorbance of the mixture was checked at 290 nm^48^. The activity of each enzyme was calculated using the following expressions.

$$\text{Enzyme activity}=\frac{\text{Absorbance }\times \text{ Total volume of the assay }\times \text{ Total extract}}{\text{ Sample weight }\times \text{Volume used for assay}}$$

### Harvesting

The study was carried out for 90 days. At the end of the growing season the representative Gladiolus plants were randomly sampled from each replicate. During the course of time, data on different parameters of growth (length and biomass of shoot and root, weight and diameter of corms) were recorded.

### Data analysis

Salt stress response indices (SSRI) were calculated initially through salt stress response index (ISSRI) for each treatment as the value of a parameter for treatment (Pt) and the value of the same parameter (Pc) at optimum condition (control) (Eq. 1) Then cumulative salt stress response indices (CMSSRI) were calculated by adding all the individual ISSRI for all the 16 measured parameters (Eq. 2)^14^

1 $$\text{ISSR}=\text{Pt}/\text{Pc}$$

2 $$\text{CMSSRI}=\text{Pt}/\text{Pc}$$

Means, standard deviations (SD) and standard error (SE) were calculated on Excel. ANOVA followed by Fisher’s protected least significant difference test (P ≤ 0.05) was used for all parameters to determine the significant effects (P < 0.05) of salinity and SA using the SATISTIX 8.1. The standard errors were presented in the figures as error bars. Principal component analysis (PCA) was performed on the correlation matrix of 20 treatments and response variables including all 16 growth and physiological attributes. SAS statistical software package version 9.3 was used to perform Principal components analysis (PCA)^49^. Additionally, $\text{CMSSRI}$ values were also used during PCA analysis to classify salt-irrigated provided with foliar application of SA.

## Conclusions

Current results demonstrated that *G. grandiflorus* is a salt-sensitive species, and a foliar spray of 100 and 150 ppm of salicylic acid could alleviate the harmful effects of salinity (2–6 dS m^−1^) through stimulating the plant’s osmolyte content, total phenolic and total antioxidant mechanisms. Overall, the results provide the first evidence to our knowledge that SA plays a role in enhancing salt tolerance in *G. grandiflorus* by modulating plant defense mechanisms. This study provides a feasible strategy for cultivating *G. grandiflorus* under saline conditions, using foliar SA as a mitigating tool against salt stress.

## Data Availability

The datasets supporting the results of this article are included in the article.
